# Towards a Decentralized Magnetic Indoor Positioning System

**DOI:** 10.3390/s151229799

**Published:** 2015-12-04

**Authors:** Zakaria Kasmi, Abdelmoumen Norrdine, Jörg Blankenbach

**Affiliations:** Institute for Computing in Civil Engineering & Geo Information Systems, Rheinisch-Westfälische Technische Hochschule Aachen University, Mies-van-der-Rohe-Str. 1, Aachen 52074, Germany; norrdine@gia.rwth-aachen.de (A.N.); blankenbach@gia.rwth-aachen.de (J.B.)

**Keywords:** synchronization, architecture, real-time clock, RTC, RIOT-OS, DS3234, TDMA, real time, periodic tasks, embedded system

## Abstract

Decentralized magnetic indoor localization is a sophisticated method for processing sampled magnetic data directly on a mobile station (MS), thereby decreasing or even avoiding the need for communication with the base station. In contrast to central-oriented positioning systems, which transmit raw data to a base station, decentralized indoor localization pushes application-level knowledge into the MS. A decentralized position solution has thus a strong feasibility to increase energy efficiency and to prolong the lifetime of the MS. In this article, we present a complete architecture and an implementation for a decentralized positioning system. Furthermore, we introduce a technique for the synchronization of the observed magnetic field on the MS with the artificially-generated magnetic field from the coils. Based on real-time clocks (RTCs) and a preemptive operating system, this method allows a stand-alone control of the coils and a proper assignment of the measured magnetic fields on the MS. A stand-alone control and synchronization of the coils and the MS have an exceptional potential to implement a positioning system without the need for wired or wireless communication and enable a deployment of applications for rescue scenarios, like localization of miners or firefighters.

## 1. Introduction

The exponential growth in information and communication technology, the increasing role of ubiquitous computing, as well as semantic-oriented information and location data mining tasks have resulted in a massive business interest in location-based services (LBS) [1,2]. Indoor localization applications and technologies enable an automatic positioning of persons or objects inside buildings and provide context-dependent information on a mobile device. Examples of indoor LBS are position assignment of products inside a warehouse and the navigation to the right platform or gate at train stations or airports [3].

The surrounding environment characterizes a position system and determines its constraints and expected performance. Theoretically, the positioning systems can be deployed both outdoors and indoors, but their efficiency differs greatly from each other, due to the fact that indoor surrounding areas raise a challenge for position finding, especially for systems based on wireless technologies, because of factors, such as: signal scattering and attenuation by reason of a high density of obstacles, multipath reflections from walls and furniture, non-line-of-sight (NLoS) and environmental changes as a consequence of opening doors and moving people [3]. Another challenge is the position finding in a harsh environment and hard industrial conditions, like a bunker, coal mine or locating a firefighter in a hazardous area.

Numerous technologies for indoor positioning have been developed over the years. In [3,4,5,6], a comparison of different technologies is provided in terms of accuracy, coverage, update rate, hardware size and cost. The main challenge of these technologies is the signal shadowing due to the presence of obstacles between the transmitter and receiver. Unlike other technologies, magnetic signals are able to pass through obstacles without significant propagation errors, even in NLoS scenarios. However, magnetic signals show a limited coverage area, since the magnetic field strengths decay rapidly with distance. Hence, large coils and high power levels are required to reach a wide coverage.

In order to design a robust positioning system in harsh indoor environments, it is of paramount importance to push the application-level data processing as deeply into the mobile station (MS) as possible and to use a localization technology, which overcomes the aforementioned limitations of existing indoor positioning systems. The processing and the evaluation of sampled data close to the source reduce the communication with the base station and minimize the energy consumption of the mobile station. The decentralized magnetic positioning system follows this strategy by designing the mobile station in such a way that the magnetic field data are at first gathered, preprocessed, synchronized and, finally, computed on-the-fly to provide the spatial coordinates of the MS.

The magnetic indoor local positioning system (MILPS) is based on direct current (DC)-pulsed magnetic signals that show no special multipath effects and have excellent characteristics for penetrating various obstacles [7]. Therefore, MILPS offers various benefits in comparison to other active positioning systems. In this paper, we propose a stand-alone localization system that enables a positioning in harsh conditions without the need for communication infrastructure, nor fixed or tedious installation. The main contribution of our work is the proposal of a decentralized control of the individual coils (anchors), as well as the decentralized synchronization of the entire system without the need of communication technology. Both the synchronization and the control of the coils and MS are based on a preemptive real-time operating system (OS) and RTCs. Furthermore, the developed work can be summarized as follows:

1. The design of a decentralized positioning system by improving the MILPS and using coil driver units (CDUs), which are based on accurate real-time clocks (RTCs). Furthermore, the MS is extended with a sensor platform, which includes a magnetic field sensor and an RTC. The MS operates independently from the CDUs, and no communication channel is required.
2. The application of time division multiple access (TDMA) for the generation of periodic, distortion-free magnetic field signals for a certain time period (e.g., 1 s). The TDMA allows the MS to distinguish between the coils (reference points).
3. The evaluation of two approaches to drive and synchronize the coils.

The remainder of the paper is organized as follows: firstly, we present the MILPS as proof of concept, then we review related works in Section 2. We introduce a new decentralized version of MILPS, as well as a decentralized synchronization approach, which is based on precise RTCs, in Section 3. We give an experimental evaluation of the system in Section 4. Finally, we conclude our paper and give an outlook on future works in Section 5.

## 2. Previous and Related Work

This section is devoted to the review of our previous work, to give a brief description of possible indoor location systems and to to overview related work on positioning based on artificially-generated magnetic fields. The emphasis is on summarizing the state of the art by focusing on the need for time synchronization.

### 2.1. Previous Work

**Figure 1:**
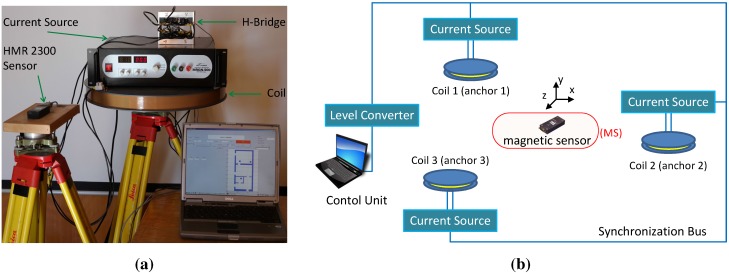
Magnetic indoor local positioning system (MILPS) platform. (**a**) Main components; (**b**) basic system overview with three coils and a mobile sensor.

The objective of our proposed MILPS is to provide a reliable and accurate indoor positioning system that covers an entire building with a minimum of infrastructure and complexity. The system consists of several coils placed inside or outside the building and a mobile sensor (*cf*. Figure 1).

**Figure 2:**
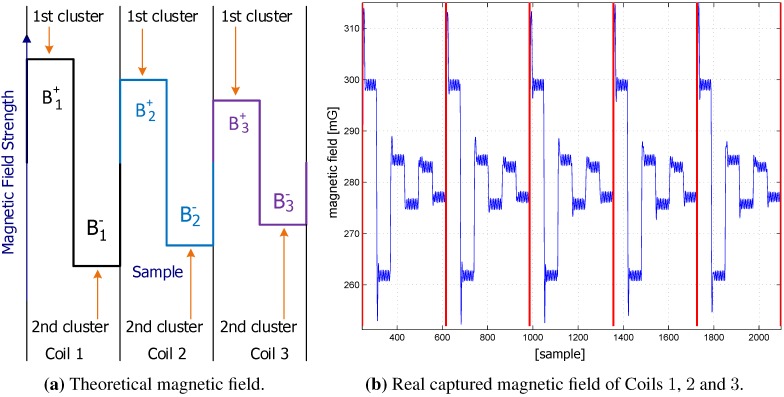
Captured magnetic fields.

The coils generate magnetic fields successively. By measuring the field components of multiple coils (at least three) and based on the coil coordinates in the building reference system, the unknown 3D coordinates of the MS can be estimated by applying the trilateration principle [8]. A simple theoretical and real example of a received magnetic field at the MS is represented in Figure 2.

As shown in Figure 2a, the direction of the electrical current of each coil is switched in polarity in order to eliminate the overlying magnetic field of the Earth and other long periodic magnetic interferences. This is achieved by computing differences between subsequent positive and negative sample clusters $\left(B_{i}^{+}-B_{i}^{-}\right)$, where $B_{i}^{+}$ and $B_{i}^{-}$ are the calculated medians of the first and second cluster, respectively. In addition, the measured magnetic field of the *i*-th coil is computed as follows: $\frac{B_{i}^{+}-B_{i}^{-}}{2}$.

A proof of concept was introduced in [9] by presenting a working prototype. The results of the measurements performed with the prototype prove the feasibility to determine the 3D position of a user or object inside a building, even in NLoS conditions. The prototype is based on artificially-generated magnetic fields and achieves a positioning accuracy of less than 0.5 m. The coils maintain synchronization through a communication link, which is implemented as a cable or a wireless link. The MS is also synchronized with the coils and, thus, can distinguish the coil fields. The synchronization acquisition at the MS is computed by using the cross-correlation between the receiving signal and a template square signal. This method is, however, limited to stationary devices, because the square wave pattern at the MS is distorted during movement. A so-called “stop and go” measurement has to be performed. That means, in order to regain the synchronization, the mobile station must regularly stop. This is unpractical in a real tracking scenario. To deal with this limitation, a complete centralized solution has been applied by sending the sensor raw data to a base station, which activates the coils and performs the position calculation. However, this method would face difficulties in a real indoor scenario, such as power consumption, low reliability of the data transfer, data congestion, when many MSs are involved, *etc*.

### 2.2. Related Work

In the past few years, numerous technologies have been evaluated for positioning and navigation tasks inside buildings. These technologies are based on several physical principles and exhibiting different performance characteristics. The physical layer can use a variety of technologies, such as ultra wideband (UWB) [10,11] or wireless LAN (WLAN) [12], which are based on electromagnetic waves. Other localization systems are based on ultrasound [13], infrared [14], radio frequency identification [15], Bluetooth [16] or computer vision techniques [17]. The main drawbacks of these systems are signal propagation errors due to attenuation, shadowing, multipath or signal delay inside buildings. Even if some technologies like UWB are more robust against the mentioned effects, it is impossible to suppress signal propagation errors completely. However, contrary to electromagnetic waves, magnetic signals are able to pass through any building material without significant attenuation or distortion and, thus, are in general very appropriate for indoor positioning purposes.

Magnetic indoor positioning systems can be classified into three categories: fingerprinting (geomagnetic), permanent magnet based and current-based magnetic positioning systems. These systems have various advantages and disadvantages, which are presented in Table 1. Examples of each category will be briefly discussed in this section.

A fingerprinting positioning system is presented in [18], which is based on the anomalies of the ambient magnetic fields. The system suggests that the ambient magnetic field may remain sufficiently stable for long time periods. Furthermore, the system can only be used for the one-dimensional location case, for example the location of a person or a robot within corridors. No coils are needed; only a three-axis magnetometer is used to achieve an object or human self-localization, but magnetic maps should be provided prior to the localization process. The magnetic maps are created for predefined paths in the initiation phase. The proposed approach can be deployed in parallel with other positioning techniques, such as range finder or machine vision methods.

The second type of magnetic positioning system performs a localization based on magnetic fields created from permanent magnets. A positioning system that is based on permanent magnets can be composed of magnetometers as reference points and a permanent magnet as a mobile target. Alternatively, multiple permanent magnets with known locations can be used as reference points to locate a mobile magnetometer. Song *et al*. present a positioning system that is composed of a cylindrical permanent magnet [19]. The permanent magnet is enclosed in a capsule and incorporated into a human body, which is located based on the measured magnetic signals from the magnetometer array. The system achieves an average position deviation of about 1.8 mm. Pham *et al*. proposes a real-time magnetic tracking system that comprises a permanent magnet and magnetometers. Based on the measured magnetic signals, the tracking is calculated on a PC with an accuracy of about 5 mm [20].

**Table 1:** Advantages and disadvantages of magnetic positioning (pos.) systems. NLoS, non-line-of-sight.

Magnetic Pos. Systems	Advantages	Disadvantages
Fingerprinting system	No infrastructure.	Acquisition of magnetic maps in the setup phase. Localization accuracy scales with the finger printing data resolution.
Permanent magnet system	High accuracy. Operating under NLoS conditions.	Restricted coverage volume (up to 1 m${}^{3}$). Mathematic models with high-order non-linear equations [3].
Current-based system	High accuracy. Operating under NLoS conditions. Not affected by reflections and multipath.	Infrastructure-based. Limited coverage area. Necessity of high power energy or highly sensitive magnetometers for coverage area extension.

The third category artificially generates magnetic signals by using coils (beacons) with known positions (reference points). The magnetic fields can be generated from coils by using pulsed direct current (DC) or alternating current (AC). Currently, commercial current-based magnetic positioning systems are designed for motion tracking and virtual reality in a number of artistic, industrial and bio-medical applications, which need a small coverage volume (typically < 1.5 m) [21,22]. In that context, three orthogonal concentric coils are used, and the magnetic sensor is connected to a central unit. The central unit activates the coils, collects the sensors’ magnetic field data and performs the localization estimation. In other words, the central unit is responsible for the synchronization between all connected components.

Sheinker *et al.* propose a 3D experimental positioning system that is based on low frequency magnetic fields. The system is composed of coils (beacons) that are excited by an AC source to generate a time-varying magnetic field [23]. In addition, the MS includes a tri-axial search-coil magnetometer, six blocks of phase lock-in amplifiers and a location calculator. The MS can distinguish between the beacons by using a lock-in amplifier, since each beacon is assigned a specific frequency. This method is similar to the frequency division multiple access (FDMA) approach. The positions are calculated on a PC based on the measured magnetic field amplitudes and phases. The system has an effective area of about 100 m${}^{2}$ and can be deployed, e.g., for robot navigation and underground cavity mapping.

De Angelis *et al.* describe the design and implementation of an indoor positioning system, which is based also on AC magnetic fields [24,25,26]. The system consists of transmitter coils that are placed at known positions and a receiver coil. Each transmitter coil is driven by a signal generator to generate an electromagnetic field, which interacts with the receiver coil by inducing a current. The position of the receiver coil is estimated based on the root-mean-square (RMS) voltage, which is measured at the receiver node. The system performs in two phases: the calibration and the trilateration phase. In the first phase, the coils and the receiver node are placed in line-of-sight conditions in an indoor environment, in order to determine the calibration parameters. In the second phase, the position of the receiver node is calculated by using the trilateration method based on the received RMS voltages from the transmitter coils.

Similar to our previous proof-of-concept, two experimental systems are introduced in [27,28], which utilize coils placed at different positions in order to reach a wide coverage area. Since these systems are still in the prototype phase, a centralized approach is also used. Prigge [27] presents a prototype system that utilizes several coils. A code division multiple access (CDMA) approach is used in order to distinguish each generated signal. A timing box generates the synchronization signal, which is distributed over a cable network to all coils and via either a wireless or a wired connection to the MS. A synchronization method is proposed in [28], which uses edge detection within the captured magnetic field signal to correct the time drift between the coils and the MS. However, this concept faces many difficulties if the captured signal is weak or the time drift is bigger than a certain amount of time, which leads to a degradation of the location accuracy. Moreover, as in our cross-correlation-based approach, the MS is not able to distinguish each single coil, because of the uniformity of the coils switching pattern [28].

Although localization systems based on magnetic fields may be valuable for cluttered environments, they are often limited by strict synchronization requirements. Accordingly, there is a need for an improved synchronization system that has low power requirements, does not require special synchronization hardware and is easy to implement in a variety of scenarios. Thus, it is necessary to utilize synchronized clocks for both the transmitting coils and the receiving MS.

## 3. Towards a Decentralized MILPS

The decentralized MILPS is based on CDUs for a stand-alone control of the coils and an MS for on-the-fly computing of the position. However, the resource constraints of the MS pose various challenges that should be taken into account during the design phase. The typical resource constraints are: restricted processing power, limited storage capacity, narrow bandwidth, short communication range and limited energy resources. The design constraints depend on the application and the environment in which the MILPS is deployed. An important design factor that should be carefully treated is the energy consumption of an MS, since the MSs are mostly battery operated. The previous considerations, like the resource constraints, the energy consumption of the MS and the system topology, provide additional inputs for the system design and architecture, as detailed in the next Section 3.1.

### 3.1. Architectural Overview

The architecture plays a major role in the performance, reliability and energy consumption of a positioning system. We distinguish between the architecture of the whole positioning system, the MS and the CDU architecture: The positioning system architecture describes the interaction and the coordination between all system components, whereby a component can be a mobile or a base station. The second specifies the design and implementation of an MS or a CDU. As data processing plays a key role in MILPS, we firstly compare different architecture approaches in this subsection. We present the architecture of the MS and the CDU in Section 3.2. After that, in Section 3.3, we give a solution for the synchronization problem in magnetic-based positioning system, which is implemented and integrated in an operating system (OS) for micro-controller (*cf*. Section 3.2.2).

There are three classes of architectures for positioning systems: central, decentralized and distributed architecture.

- Centralized Architecture: This is the most commonly-used architecture, in which the sensors or the MSs exclusively communicate with the base station, which usually possesses more processing power, storage capacity and energy resources than the MSs. The MS modules deliver data to the base station, such as raw sensor data or the results of a signal processing step; normally, with the compression or reduction of the data. The individual MSs have no knowledge about the semantics of the gathered data that are transmitted to and interpreted in the base station. The advantages of a centralized architecture include the usage of lightweight and low-cost MSs, since the whole complexity is shifted to the base station; a high position resolution and a full central overview of the observed phenomenon (e.g., event). The disadvantages comprise single point of failure (e.g., the base station failure), which is not tolerable in a secure scenario; poor performance with a large number of MSs; the system can get into energy starvation in case of continuous communication between the MSs and the base station; and the power-saving techniques are difficult to implement.
- Decentralized and Distributed Architecture: In this architecture, data processing and position computation occur in the mobile station, and no data, except the result of a position finding, are sent to the base station. Furthermore, in a distributed MS network, which is also referred to as collective evaluation [29], the MSs can exchange data, in order to collectively achieve and respectively augment the efficiency and the precision of a positioning task [30]. This can be important in the case of using distributed localization algorithms [31]. The distributed approach also favors cooperative sensing and positioning when multiple MSs are present. The decentralized and the distributed architecture exhibit the following advantages:
  1. Scalability: the computational load and the communication overhead at each MS do not depend on the MS number.
  2. Robustness: the system is not affected if an MS or some MSs fail, since no node has a designated role and all nodes can perform the data processing and transferring tasks.
  3. Energy-awareness: the data traffic between the base station and the MSs is reduced, since this architecture supports on-board or in-network processing, saving both link bandwidth and node energy, which are critically-constrained resources [32].
  The drawbacks of the decentralized and distributed architectures are: the MSs have no global knowledge about the network topology or about a phenomenon. Components of the distributed architecture only know about connections in their own neighborhood, which requires a robust and self-healing network. Since the aim of the decentralized or the distributed architecture is on the mobile station or in-network processing, respectively, the use of lightweight and low-cost MSs is not possible. The MS should have a micro-controller unit (MCU) with more computational power and memory capacity, as well as an efficient application layer and, in the case of a distributed evaluation, a communication protocol stack. The distributed position evaluation is not the focus of this paper. We use the decentralized architecture due to the scalability, robustness and energy-awareness and on-the-fly capability of the MS.

### 3.2. Mobile Station and Coil Drive Unit Architecture

Expanding on the principles of MILPS and the architectural view presented in the previous subsection, the focus is shifted from the system topology aspects to the MS and the CDUs’ design aspects. Based on the general discussion of architectures, MILPS principles and the synchronization problematic of the system, we now present an exemplary platform for a decentralized and synchronized magnetic positioning system. This platform is employed in MILPS, whereas the mobile station and the anchors are equipped with real-time clocks, and the MS additionally incorporates a magnetic sensor. The MS, as well as the CDU are designed in a layer-based architecture.

**Figure 3:**
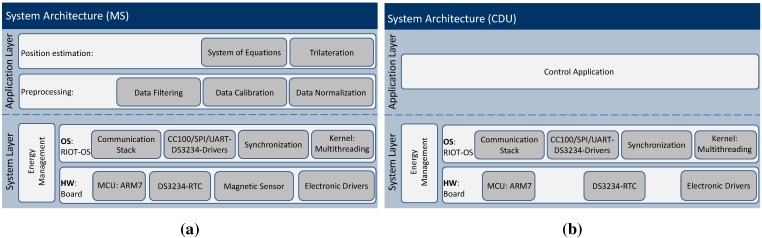
System architecture of (**a**) the mobile station (MS) and (**b**) the coil driver unit (CDU).

As illustrated in Figure 3, the system architecture is divided into two layers: the application layer (AL) and the system layer (SL). The SL includes the hardware board, the power unit and the OS. The RIOT-OS [33] is a tickless real-time OS that supports multithreading and priority-based preemptive multitasking (*cf.* Section 3.2.2).

The AL is the highest level of the MS or CDU, which follows a modular-based architecture that ensures the portability and the extensibility of the system. In the case of the MS, it is subdivided into two sublayers: the preprocessing and position computing layers (*cf*. Figure 3a). The first sublayer includes the data filtering module, which removes statistical outliers from data delivered from the system layer. The second preprocessing module provides a calibration routine to correct inaccuracies of the magnetic samples. The top level sublayer represents the algorithmic core that computes the position on the MS. In the case of the CDU, the AL includes an application for controlling the coils. Both ALs incorporate a command shell for the interaction with a user or an application by using the serial interface.

#### 3.2.1. Hardware

**Figure 4:**
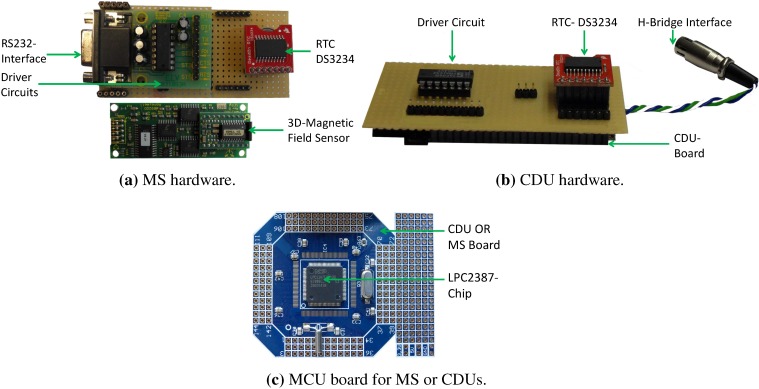
MILPS hardware.

The hardware comprises four subsystems: the power unit supplying the sensor board with the energy, the micro-controller unit (MCU), the sensing unit and the driver circuits. The MCU forms the core of the system controlling the other three subsystems. The MCU is based on an ARM7 core, operating at 72 MHz and has a memory capacity of 96 KB RAM and 512 KB ROM (*cf.* Figure 4c).

The sensing unit includes the 3D-magnetic sensor HMR2300, which offers a sampling rate of up to 154 Hz and a range of ±2 gauss (G), with a resolution up to 70μG [34]. The sensing unit can be extended with additional sensors, like a 3D-accelerometer or gyroscope sensors, in order to support a navigation scenario in indoor environments. The hardware of the MS and the CDUs is illustrated in Figure 4.

The driver circuits enable the MS boards to interface with the magnetic sensor (*cf*. Figure 4a) and the CDU boards to drive the coils, which generate a square-wave magnetic field signal, as illustrated in Figure 2. The coils are controlled via a CDU through a solid-state relay (SSR) unit, which is interfaced with a driver circuit (*cf*. Figure 4b). The SSR-Unit consists of four relays that have a maximum turn-off/on time of 300μs/1.0μs. The four SSR switching elements form an H-bridge, in order to control the voltage polarity and to enable a galvanic isolation between the CDU and the high voltage load circuit without the use of mechanical parts [35].

#### 3.2.2. Operating System: RIOT-OS

We now switch our focus from the hardware view to the operating system issues. As shown in Figure 3, the hardware configuration affords, on the one hand, the MS sensing, storing and processing of sensory data and, on the other hand, the CDUs switching the coils. The tasks on the MS and the CDUs run quasi-parallel [36], performing both sampling the magnetic data and the synchronization task on the MS. In this case, a multithreaded operating system (OS) is needed, which represents the upper sub-layer of the system layer and relies on the bottom of the hardware sub-layer (*cf*. Figure 3). The multithreading allows a better use of the system resources, such as overlapping of I/O operations and computing, as well as the implementation of more responsive applications [37]. Based on the characterization of operating systems, we compare various OSs, in order to choose an appropriate OS for MILPS. An OS can be essentially characterized by the following key design issues: (*i*) the kernel structure, which can follow a monolithic model, layered approach or microkernel paradigm; (*ii*) the scheduler is the OS part deciding which task to run next; and (*iii*) the programming model provides an abstract view of the hardware for the application developers by hiding the hardware complexity and defining the context in which the tasks are executed [33].

Resource-constrained devices, such as microcontrollers, are characterized by a scarce resource of energy, limited storage capacity and low computation power. The OSs for resource-constrained devices vary in the architecture, the programming model, scheduling, memory management and protection, communication protocol and the real-time support [38]. Examples for these operating systems are: FreeTOS [39], TinyOS [40], Contiki [41], MANTIS [42], Nano-RK [43] and LiteOS [44].

Since FreeTOS, TinyOs and Contiki are the most popular and dominant open-source OSs for memory-constrained devices, we focus on and compare them with RIOT-OS. The comparison is summarized in Table 2.

**Table 2:** Comparison of operating systems. FIFO, first-in first-out. TinyOS, TOS

OS	Architecture	Scheduling	Programming Language	Programming Model
FreeRTOS	Monolithic	Round-robin preemptive and cooperative	C	Threads
TinyOS	Monolithic	FIFO	nesC	Primarily event driven, support for TOSThreads
Contiki	Modular	Event based	C with some constraints	Protothreads and events
*RIOT-OS*	*Microkernel*	*Tickless, preemptive scheduling with priorities*	*C and C++*	*Threads*

Although the FreeTOS is convenient for the most lightweight microcontrollers, FreeRTOS does not support low power management features, like most real-time operating systems, due to the fact that the energy-savings of modern MCUs is platform dependent [45,46]. Furthermore, they use periodical timer interrupts to manage the timers and the system time. The MSs must be able to act long enough for months or even years on battery supply, to accomplish their propose.

TinyOS is based on a monolithic kernel, while Contiki kernel is close to a layered approach. The scheduling in both OSs is purely event driven and uses a simple first-in–first-out (FIFO) strategy. The programming model in both OS kernels is event driven, and all tasks share the same context. TinyOs and Contiki exhibit some developer-unfriendly issues: (*i*) TinyOS and Contiki do not support standard multi-threading and real-time applications; (*ii*) proto-threads are pseudo-threads that enable the implementation of blocking threads in Contiki, but they have some limitations, such as local variables are not preserved, as well as thread functions are not reentrant; and (*iii*) the programming language nesC is not apprentice friendly [47].

To remedy the drawbacks observed in the previous discussion, we decided to use RIOT-OS [33,48] developed at the “Freie Universität Berlin”. RIOT-OS is based on a microkernel architecture, which is inherited from FireKernel [45] and was deployed for a rescue scenario to track and monitor fire fighters. In order to fulfill severe real-time requirements for hard industrial or emergency scenarios, the micro-kernel provides a zero-latency interrupt handling and prioritized threads with a minimum context-switching time. Regardless of the system load, the maximum interrupt latency of the LPC2387 MCU amounts to 50 cycles (700 ns); and the maximum context switch time outside an interrupt service routine (ISR) is 72 cycles (900 ns) (*cf*. Table 3).

**Table 3:** Measured latencies of the LPC2387 MCU with a 72-MHz operating frequency; source: [45].

Interrupt Types	Cycles	Time in μs
Interrupt latency	50	0.700
Context switch outside an ISR	72	0.990
Context switch	600	8.4
Inter-process communication (IPC) delay	1300	18

A tickless scheduler is implemented, in order to achieve a maximum energy savings and to support deep-sleep mode by all resource-constrained MCUs. Since most schedulers wake up periodically to enable the switching between tasks, their behavior is dependent on periodic timers, which is not desirable for the energy awareness. Furthermore, the most constrained devices cannot be woken up from a timer interrupt, but only from external interrupt sources. In this manner, periodical timer interrupts prevent the deep-sleep mode, and this leads to excessive power consumption over the entire run-time.

#### 3.2.3. Device Drivers

Based on the architecture of the RIOT-OS, we develop and integrate device drivers for the DS3234 real-time clock, the HMR2300 magnetometer, the serial peripheral interface (SPI) bus and the universal asynchronous receiver transceiver (UART) controller. These software driver components build the driver module and are part of the system layer (*cf*. Figure 3). Due to the support of fast interrupt handling (low interrupt latency) and multi-threading, the RTC and HMR2300 drivers run in concurrent threads, thus enabling the synchronization of the sampled magnetic data on the MS. Because of the energy efficiency, RIOT-OS enables the development and deployment of energy-aware applications on resource-constrained devices. Further energy savings can be achieved by using MCU-specific power management techniques, which improve the lifetime of the MS. The modular structure of RIOT-OS allows not only the development of a magnetic-based positioning system, such as MILPS, but also the deployment of localization systems that are based on other technologies. The modularity and the developer-friendly features encourage the reusability of common modules, e.g., filtering algorithms. The reusability of source code is improved by defining clear interfaces between the individual layers and sub-layers. Therefore, MILPS can be easily extended with other technologies: by fusing data from different types of sensors, it is possible to compensate for the shortcomings of a single technology, such as coverage gaps, or to improve the positioning performance.

### 3.3. Synchronization

The synchronization plays a key role for MILPS, since the coils are activated in successive time slots (TDMA), and the MS has to classify the sampled magnetic data into the proper coil at the corresponding time. In general, a vital element for proper operation is a reliable clock source, which is essential for electronic systems, like microcontroller, microprocessor or discrete logic. Since oscillators are the basis of the MILPS source clocks, they will be discussed along with timing and synchronization on resource-constrained devices in the next Section 3.3.1 and Section 3.3.2. Furthermore, we introduce a decentralized synchronization approach based on a high precision real-time clock (RTC), as well as two synchronization methods of the CDUs.

#### 3.3.1. Stability and Accuracy of Oscillators

The quartz crystal is the core component of crystal oscillators, which is affected by factors, like temperature, humidity, power supply voltage and mechanical shock [49,50]. Furthermore, the frequency and stability can be negatively impacted by the following: (*i*) Aging is a gradual change in frequency over a long period of time, due to electromechanical effects, like mass transfer and stress in the quartz; (*ii*) Short-term instabilities are standard deviations of the fractional frequency fluctuations for a specific averaging time that are random in nature and are referred to as noise; (*iii*) Phase noise is the random fluctuations in the phase component of the output signal [51]; and (*iv*) Temperature drift means that the resonant frequency of the crystal varies depending on the ambient temperature; consequently, high temperatures affect the nominal frequency, which can reach a deviation of up to a few tenths of parts per million (ppm) of seconds [51].

#### 3.3.2. Timing and Synchronization on Resource-Constrained Devices

Modern microcontrollers incorporate an internal RC oscillator, which is inaccurate and sensitive to the supply voltage and temperature variations. Therefore, an external crystal oscillator is used for improved stability, frequency accuracy, low power consumption and the flexibility of a wide choice of frequency values [52]. The most common oscillator configurations for microcontrollers are the Pierce and the Colpitts circuits [53,54].

Resource-constrained devices support timers with high resolution (up to 1 μs), which are convenient for time measurement or for performing a task during a given time interval, but they do not represent an absolute time. The absolute time plays a key role for the synchronization of MILPS, since periodic distributed tasks rely on it. A further requirement is the kernel preemption, enabling application processes to preempt the kernel. Conversely, the synchronized tasks can be strongly affected by other activities, such as communication tasks, which do not support preemption, or when the system is exposed to a heavy load. Nevertheless, the source clock remains the weakest part of the system due to the temperature drift or aging (*cf*. Section 3.3.1).

#### 3.3.3. Synchronization Scheme Based on Real-Time Clocks

**Figure 5:**
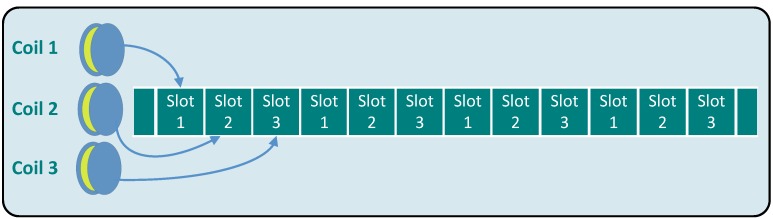
System synchronization (TDMA).

Based on the general discussion of the oscillators’ stability and synchronization on resource-constrained devices, we now present a decentralized synchronization mechanism, which implements the periodic control of the coils and the MS using the TDMA scheme (*cf*. Figure 5). The MS is synchronized with the CDUs without the need for a synchronization bus or any kind of communication.

The goal is to generate time slots with a certain period of time (e.g., 1 s) or multiples thereof, with a tolerance range of ±20 %, which must not be exceeded during the total operating time of 2–3 h (e.g., the period of a rescue operation). The core of our method is based on the DS3234 low-power and accurate RTC with the accuracy of ±2 ppm or ±3 ppm for the temperature ranges from 0°C to 40°C or from -40°C to 80°C, respectively. The performance of the DS3234 is achieved by the combination of a temperature compensated crystal oscillator (TCXO), which provides a high level of temperature stability, and the RTC [55].

The built-in TCXO offers an accurate and stable reference clock, which keeps the accuracy of the RTC within ±2 min per year in a temperature range from -40°C to 80°C [55]. In addition, the TCXO provides a square-wave with four programmable frequencies from 1 Hz to 8.2 kHz and a battery backup unit in order to continuously keep the time and settings in 256 bytes of SRAM.

For a typical deployment, MILPS operates in three distinct phases:

1. The initialization phase: Both the RTCs of the CDUs and the MS are set to the same time. This phase is initiated by a user with the help of a serial splitter or wireless connection: the RTC is integrated in the CDU and the MS via the SPI-interface. Once the initialization is complete, the system enters the operating mode.
2. The operating mode: In this mode, each coil’s CDU is assigned to a fixed duration slot, in which the coils are activated. Like TDMA, the time slots are cyclically organized (*cf*. Figure 5). Simultaneously, the MS acquires the magnetic data from the HMR2300 sensor, which can be assigned to the source coils by means of the RTC and the predefined time windows. Based on the RIOT-OS, which provides a preemptive kernel scheduler and a fast interrupt handling, the data sampling and the time division run in different threads. Despite the clock drifts of the temperature-compensated RTCs in this phase, the distance measurement is only affected if a certain time is elapsed. The maximum time threshold, which affects the distance measurement, is examined in the experimental part and is in the order of 80 h.
3. The resynchronization phase: The clocks of the MS and the CDUs are reset to the same time, before the maximum time threshold elapsed.

#### 3.3.4. Coil Synchronization Schemes

**Figure 6:**
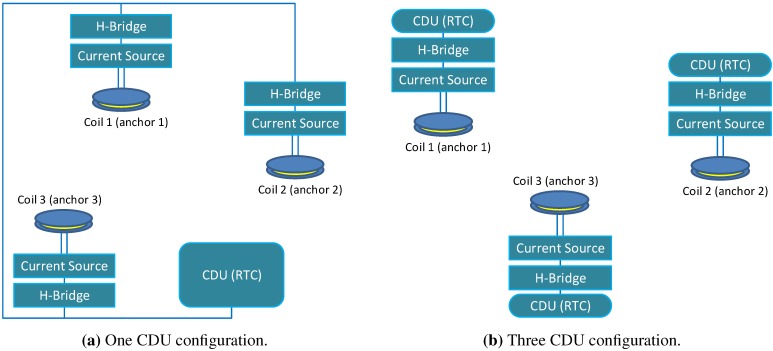
Synchronization configurations for three coils.

Independent of the synchronization of the MS, the coil synchronization is based on RTCs and can be used in two configurations:

1. The one CDU configuration in which the coils are driven by the same CDU. This configuration is less sensitive to the clock drifts, because the drift occurs only between two clocks: the CDU and MS clocks (*cf*. Figure 6a).
2. The N-CDU configuration in which each coil is driven by a separate CDU. This synchronization is more sensitive to the clock drifts than the one CDU configuration, since the time drift can occur between each CDU- and MS RTC and between the CDU RTCs in the neighboring time slots (*cf*. Figure 6b).

## 4. Experimental Evaluation

In this section, we present the results from our preliminary experimental evaluation of the accuracy of the DS3234-RTC clocks in a real indoor environment. Subsequently, we validate the correlation of the magnetic field data, which are generated from three coils, with the magnetic values that are acquired from the MS. Furthermore, we investigate the impact of the clock drift on the calculated distances and position using the measured coil magnetic field at the MS.

### 4.1. Clock Drift Evaluation

Firstly, we measure the drift between two DS3234-RTCs by using a digital sampling oscilloscope (DSO) and quantifying the time deviation over a period of three weeks in an average ambient temperature of about 20.5°C.

**Figure 7:**
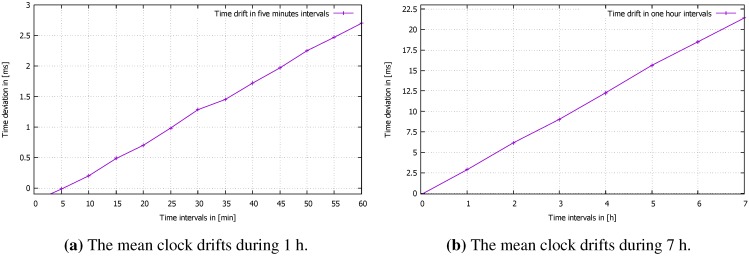
Time deviations by clocks.

According to our measurements, the DS3234-RTCs have an average drift time of about 2.7 ms per hour (0.75 ppm), which corresponds to a 0.4 sample deviation within an hour by the maximal sample rate of 154 sample/s of the HMR2300 magnetometer. Figure 7 represents the average time drift within one and seven hours between two DS3234-Clocks, respectively, whereby both figures show a time drift, which is different from zero by t=0 s. Although the RTCs are set to the same time by t=0 s, they show an average time drift from one up to two tenths of milliseconds, due to the time delay during the initialization phase.

According to the measurements of the RTCs, the magnetic field data on the MS would have an average sampling error of 30 samples in three days, which corresponds to ±19% of the coil cluster time (*cf*. Figure 2a), and it will be acknowledged in the results of the second experiment. The observed average drift time of about 2.7 ms does not differ significantly from the theoretical drift of about 3.0 ms, which is calculated by using Equation (1) [56]:

(1) $$ERR\left(ppm\right)=-0.042*{\left(25-T\right)}^{2}$$

whereas ERR is the theoretical crystal frequency error and *T* is the operating temperature. Theoretically, in the worst case, the average sample error after an hour is 0.4 samples, which can lead to approximately ±20% of each coil cluster signal after three days. The RTC time drifts away from the real time in a different direction and at a different rate (slow or fast), which depends on the factors mentioned in Section 3.3.1 and other subtle environmental variables.

### 4.2. Synchronization Evaluation

**Figure 8:**
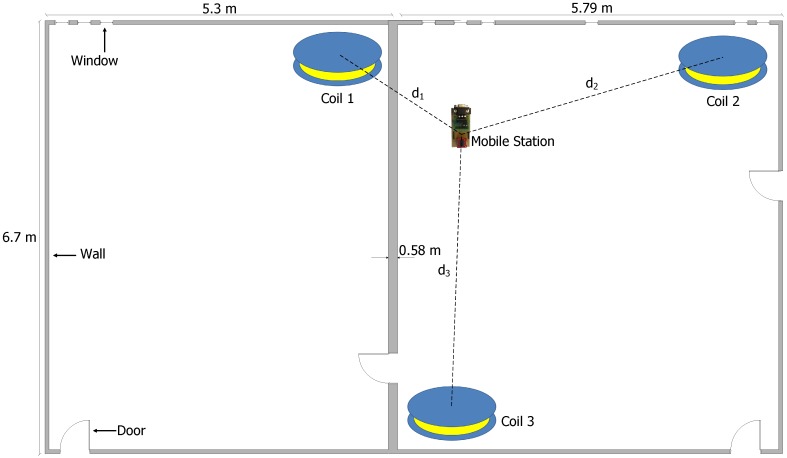
Experimental setup for distance measures between an MS and three coils.

Secondly, in order to examine the synchronization on the MS and the impact of the clock drift on the calculated distances and position, we setup a real-world experiment with three CDUs and one MS, which gathers and synchronizes the magnetic data on a fixed position (*cf*. Figure 8). The true distances between the MS and coils 1, 2 and 3 are 2.15 m, 3.45 m and 3.9 m, respectively. The coils are placed in the corners of two rectangular rooms with a 6.7×5.3 m${}^{2}$ and 6.7×5.79 m${}^{2}$ surface. The rooms are separated by a wall, which is 0.58 m thick (see Figure 8). The three coils are controlled in two different ways, via one CDU or three CDU units, in order to compare the two CDU configurations with respect to the clock drift, as well as the distance and position deviation. Furthermore, the synchronized data and the calculated distances are logged in real time on the MS, than transferred and subsequently evaluated on a PC. At the beginning of the experiment, the clocks are initiated once. Afterwards, all clocks are not resynchronized during the experiment, in order to provide the maximum time threshold affecting the distance measures. The distances $d_{1}$, $d_{2}$ and $d_{3}$ between the MS and coil 1, 2 and 3 are respectively calculated for a period of 104 h every four hours.

**Figure 9:**
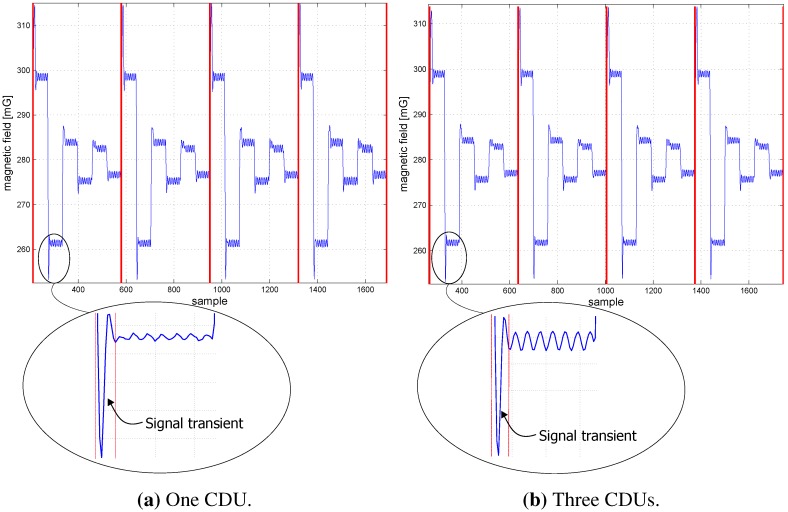
The magnetic field at the beginning of the experiment and signal transients.

**Figure 10:**
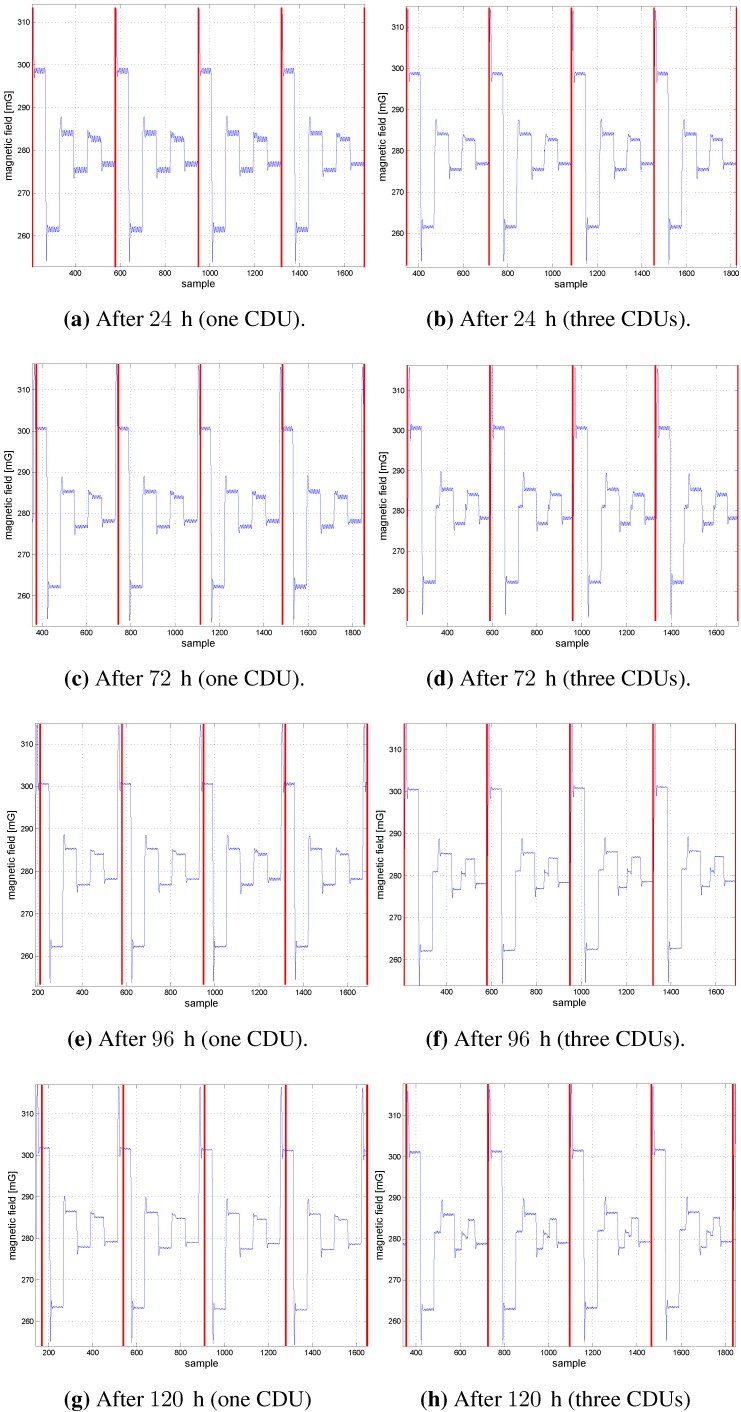
The magnetic field samples of three coils (one CDU and three CDUs).

The vertical red lines in Figure 9 and Figure 10 represent the beginning of coil 1’s first cluster, which is detected from the MS. Figure 9 shows the coil clusters after the initiation of the CDU units and the MS in the one and three CDU configurations. Signal transients occur at the beginning of each cluster, due to the switching of inductive loads (coils). Figure 10 shows the coil clusters for both one and three CDU configuration after 24, 72, 96 and 120 h. The coil’s cluster signals are free of distortion after one day (*cf*. Figure 10a,b). After three days, the sampling signals are distorted by using the three CDU configuration (*cf*. Figure 10d,f,h); in contrast, the signals remain unchanged and distortion free by the one CDU configuration (*cf*. Figure 10c,e,g). The signals are free of distortion, since the coils are controlled via one single CDU. By contrast, the signal distortion of the N-CDU configuration is due to the superposition of magnetic fields originating from coils with neighboring time slots (e.g., time slots 1 and 2). A signal superposition occurs when the CDU of a neighboring time slot does not begin or terminate the coil activation at the right time. After 96 or 120 h, strong signal distortions arise, which are shown in Figure 10f,h, respectively. Although the one CDU configuration does not exhibit any cluster distortion, the time drift between the CDU and the MS RTC rises steadily with time and reaches a time drift of about a third of the cluster time after 120 h (*cf*. Figure 10g). The time drift between the CDUs and the MS RTC is marked by the red vertical lines.

**Figure 11:**
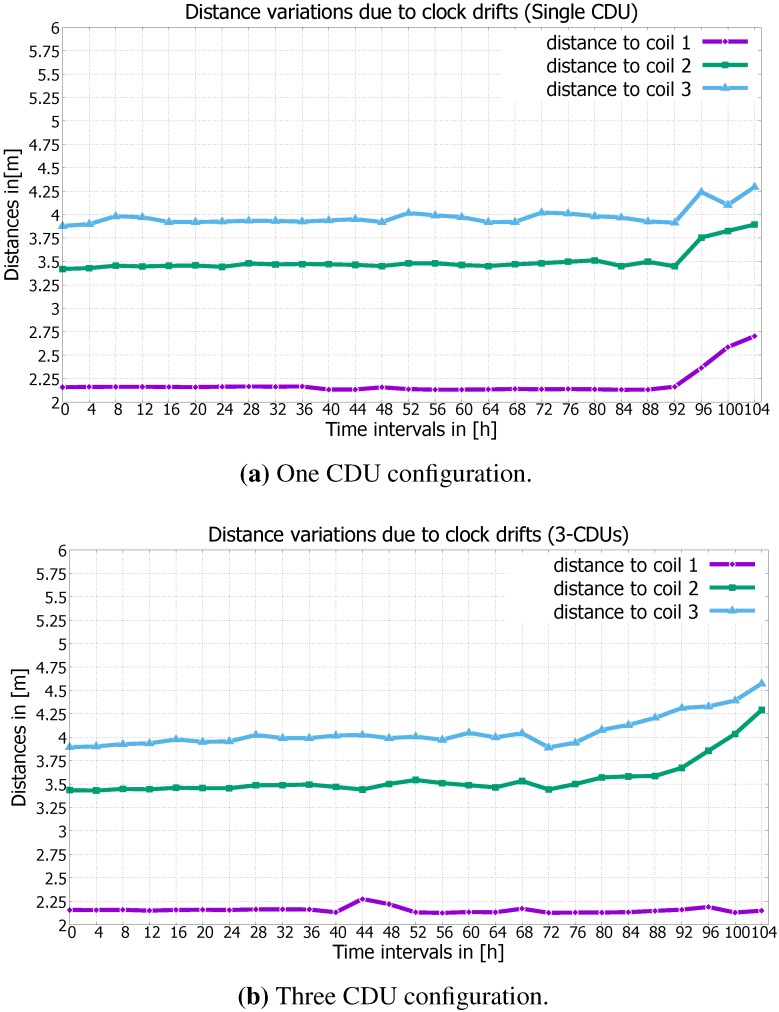
Comparison of distance deviations.

The calculated distances $d_{1},d_{2},d_{3}$ between the coils and the MS remain relatively constant in both CDU configurations after a time lapse of 72 h and have an error up to a few centimeters (*cf*. Figure 11), since the one and three CDU configurations feature small time drifts within this time interval, and the clusters are not strongly distorted in the three CDU configuration (*cf*. Figure 10d). Furthermore, the gathered values of the magnetic field are ignored from the MS for the transient period (*cf*. Figure 9), which consequently decreases the effect of the time drift between the RTCs. By the one CDU configuration, the distances stay relatively stable in the time interval from 72–92 h, whereby the maximum deviations of the calculated distances $d_{1}$, $d_{2}$ and $d_{3}$ are about 21, 30 and 34 cm, respectively (*cf*. Figure 11a). In contrast, during the same interval, the maximum deviations of the calculated distances $d_{1}$, $d_{2}$ and $d_{3}$ are about 4, 40 and 43 cm by the three CDU configuration, respectively (*cf*. Figure 11b). As expected, the N-CDU configuration generally exhibits a larger distance deviation compared against the one CDU configuration, since the N-CDU configuration is subject to (N+1) error sources: N-CDU and one MS RTCs. This leads to a false synchronization between the CDU RTCs and the MS RTC, as well as to a superposition between the coils’ magnetic fields of successive time slots. Furthermore, due to signal transients, five values of the captured magnetic field data, which correspond to a 40 ms guard time, are discarded from the beginning and the end of each cluster. Thus, the calculated distances and positions has not yet noticeably affected by time drift. However, particularly after 92 h, the drift is significantly bigger than 40 ms and leads consequently to higher errors in the calculated distance and positions.

The distance $d_{1}$ in the three CDU configuration remains almost stable after a time lapse of about 104 h (*cf*. Figure 11b); it presents an exception, since the MS and the CDU RTC of the first coil drift coincidentally in the same direction and nearly with an equal rate. Therefore, there is no time drift between the the MS and the first CDU RTC (see the vertical red lines in Figure 10b,d,f,h). Moreover, the first cluster remains distortion free, since by coincidence, the RTC of the third cluster (the third time slot) ceases early and the RTC of the second cluster begins late to deactivate or activate the coils, respectively.

**Figure 12:**
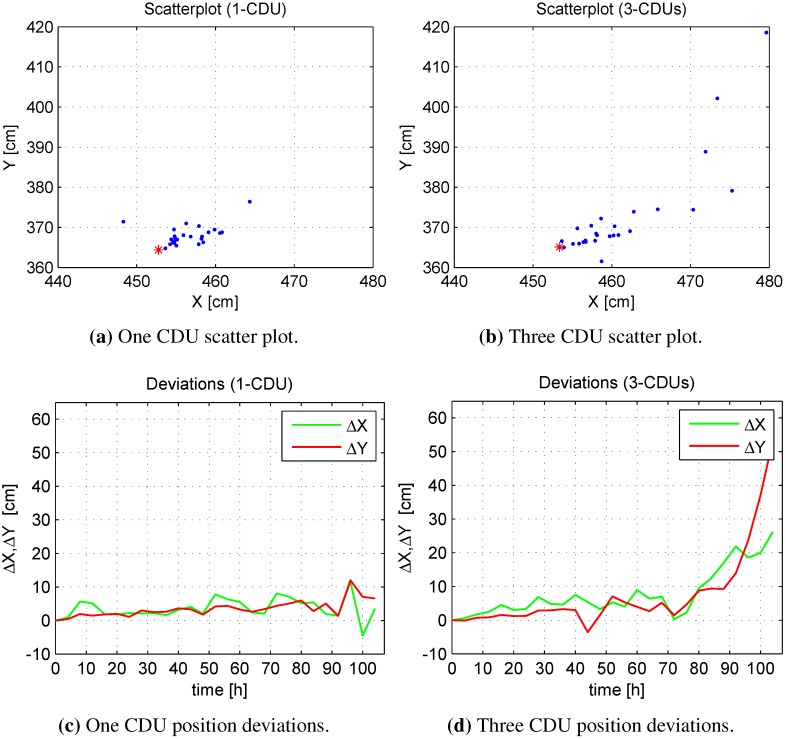
Comparison of position deviations.

The MS position is determined by using the distances $d_{1}$, $d_{2}$ and $d_{3}$ from the MS to the coils. The distances are calculated on the MS and transmitted in real time to a PC, in order to compute the MS position deviations in regard to the time drift for both CDU configurations. Figure 12 shows the position deviations of the MS for the one and three CDU configurations, whereby the one CDU demonstrates less variation than the three CDUs in relation to the calculated position (*cf*. Figure 12a,b).

The three CDU exhibits more variance than the one CDU in the x- and y-components of the MS coordinates after a time lapse of about 80 h (see Figure 12c,d), since this configuration depicts more distance deviations due to cluster distortions and the time drift to the MS compared to the one CDU configuration. The three CDU configuration causes more distance deviation than the one CDU configuration and, hence, diminishes the precision of the calculated position, particularly after a time of about 80 h.

## 5. Conclusions and Outlook

In this article, we present a decentralized approach for a magnetic indoor positioning system, which is synchronized by temperature-compensated RTCs. We demonstrate that the realization requires a reliable clock source in terms of temperature drift and a preemptive OS. Furthermore, we suggest two methods to control the coils: the one and N-CDU configuration. Both synchronization configurations are based on the TDMA approach and can be deployed at least for three days after the RTCs are initialized. In order to extend the operating time of the MS, we deploy a tickless micro-kernel and we use a decentralized evaluation of the sensing data. The decentralized architecture enables us to push the application data deeply into the MS, and so, communication with a base station can be avoided. The stand-alone controlling of the coils, as well as the on-the-fly computing of the position on the MS increase the potential to implement a system for harsh conditions. In such scenarios, the wireless communication can be unreliable or severely affected. As future work, we will compare further positioning algorithms in terms of complexity, memory capacity requirements, convergence rate and their suitability for resource-constrained MS.
